# Supplementary material for: Are carbon emissions trading and green financial instruments synergistic? -Comprehensive quantitative research based on content analysis
Source: PLoS One. 2024 Mar 7;19(3):e0298601. doi: 10.1371/journal.pone.0298601 (PMC10919601; doi:10.1371/journal.pone.0298601)
Supplement: S1 Data — (ZIP) [file pone.0298601.s001.zip › Data/地级市Z_F工作报告.zip!/║■─╧/│ñ╔│╩╨/└┤╫╘╣1⁄2╓┌║┼í╛┬φ┐╦╩2╛▌═°í┐_2004.docx]

各位代表：

　　现在，我代表市人民政府向大会报告工作，请予审议，并请各位政协委员和列席人员提出意见。

　　一、２００３年工作的回顾

　　２００３年是本届政府的开局之年。在省委、省政府和市委的正确领导下，全市人民以“三个代表”重要思想为指导，认真落实党的十六大精神，始终把发展作为第一要务，面对突如其来的非典疫情，前涝后旱的自然灾害，同心同德，埋头苦干，战胜困难，励精图治，圆满地完成了市十二届人民代表大会第一次会议提出的各项目标任务，全市出现了人心振奋，和谐发展，蓬勃向上的崭新局面。

　　（一）结构调整取得显著成效，国民经济保持了良好的发展势头。

　 　全年完成国内生产总值９２８.２亿元（预计数，下同），增长１４％；全市财政总收入达１００.０２亿元，增长３２.５％，完成地方财政收入７５亿元，增长３６.４％；一、二、三产业分别增长４.８％、２０.３％和１０.５％，三次产业结构为８.９∶４２.５∶４８.６；工业完成增加值２６８.５亿元，规模工业实现利润３８亿元，均创历史新高；在稳定粮食生产的前提下，全市增加经济作物种植面积１７万亩，养殖业产值占农业总产值比重达４６.３％；乡镇企业完成产值８４０亿元，增长１５％；高新技术产业实现产值３９１亿元，增长２５％；社会消费品零售总额４５２亿元，增长１２.６％。２００３年我市经济发展呈现的良好态势，标志着全市经济运行进入了新一轮发展增长期！

　　（二）基础设施建设快速推进，城乡面貌发生了深刻变化。

　 　全年共完成全社会固定资产投资４８０.６亿元，增长３４.４％，其中基本建设投资２０１亿元。完成重点建设项目３５个，新增城市道路９８公里。霞凝港一期工程提前竣工；五城会场馆及配套设施按期完工并交付使用；机场高速、韶山北路、湘江路南北段拓改等工程相继完成；建成了贺龙体育场等设计新颖、特色鲜明、文化内涵深厚的标志性建筑。第一污水处理厂扩建后正式投入运行，黑糜峰垃圾无害化处理场建成启用。开展了“城市管理综合整治年”活动，拆除违章建筑９０余万平方米；投入３亿元资金，对城区实施了

净化、美化、亮化、绿化工程。全市投入资金１５.８亿元，新建、改建县乡公路３３９公里，完成村级公路硬化６１４公里；投入水利建设资金８.２亿元，完成水利工程６万余处；小城镇建设稳步推进，完成投入４１亿余元。省级园林城市建设通过验收，城市总体规划修编已经国务院批准并付诸实施。特别令人欣慰的是，我市的城乡建设，尤其是市区建设，在全市人民的大力支持和热情参与下，“三年大变样”的目标顺利实现。我们以一流的组织、一流的场馆、一流的环境、一流的服务履行了对举办第五届全国城市运动会的承

诺，赢得了国内外宾朋的普遍赞赏，充分展示了长沙的新形象和长沙人民创造新生活的良好精神风貌！

　　（三）改革开放不断深化拓展，开放型经济发展迅速。

　 　全年完成市属国有企业改制５８户，改制面累计达７０％，理顺职工劳动关系４.３万人。商贸企业全面完成改制。投融资方式不断创新，推出了重点项目集合委托贷款模式。新增融资担保机构１３家，为中小企业提供贷款担保５.３亿元。积极推行部门预算、基建预决算审查，政府采购范围和规模不断扩大。干部人事制度改革不断推进。建立和推行行政问责制度，行政管理方式改革取得新进展。完成进出口总额１９.８亿美元，增长１９％；“民交会”、“深洽会”、“欧洽会”等重大经贸活动取得丰硕成果；实际到位外资、引进市外境内资金分别达５亿美元和２３０亿元，分别增长５２.４％和７８％；全市有２２家企业走出国门。成功举办了第二届世界华文传媒论坛和首届中国长沙国际旅游论坛。

　　（四）科技、教育、文化协调发展，社会各项事业全面进步。

　 　实施科教兴市战略，科技对经济增长的贡献明显增强。应用研究得到加强，形成了一批核心技术和自主知识产权成果，全年专利申请１８６０件。基本完成了以区、县为主的义务教育管理体制调整，在全省率先开通了教育信息网络平台，市区中小学布局调整取得了阶段性成果，长沙大学专升本工作进展顺利。文化事业繁荣活跃，成功举办了“映山红”民间戏剧节，电视音乐《阳光乐章》和广播剧《空房子》分别获广电部星光奖和全国优秀广播剧一等奖。群众性体育活动丰富多彩，竞技体育取得新的成绩。在第五届城运会上，夺得１９枚金牌，取得了综合排名第五的好成绩。医疗卫生救治和防疫能力全面增强。面对“非典”冲击的严峻局面，全市上下众志成城，雷厉风行，群策群力，排除万难，实现了“非典”疫情零感染，夺取了防治“非典”的重大胜利。人口与计划生育工作被评为省级模范市。大气、粉尘污染得到了有效防治，全年空气质量优良率达７０％。加强了国土管理，土地、山体和水资源得到了有效保护。实施人才强市战略取得了新成效。双拥工作扎实开展，第三次荣获“全国双拥模范城”。工商、税务、药监、海关、质监工作进一步加强，荣获全国质量兴市先进市。审计、统计、物价、人防、地震、气象、水文、消防、档案、台办、新闻出版、民族宗教、市志等工作成绩突出，工会、共青团、妇联、老龄委、残联、关协和侨联等工作取得新进步，为我市经济和社会的全面发展做出了新的贡献。

　　（五）民主法制建设加强，政治文明和精神文明建设富有成效。

　 　切实尊重和保障人民的政治权益。自觉接受人大及其常委会的法律监督、工作监督和政协的民主监督。全年办理人大代表议案、建议、批评、意见３０５件，办理政协提案３３６件。提请市人大常委会审议通过并颁布了《长沙市标准化管理条例》，成为全国唯一制定标准化管理地方法规的省会城市。制定并公布政府规章６件，审查政府规范性文件３５件。坚持依法治市，继续开展“四五”普法活动，领导干部学法用法的自觉性和人民群众的法律意识增强。政务公开范围和领域进一步扩大，不断加强审计监督和行政效能监察，开展了行风评议，全面强化了行政责任，政府工作作风进一步好转，凝聚力、感召力明显增强。信访工作及时排解了一些群众反映的难点和热点问题。基层民主建设成效显著，６４个社区在全省率先实行直接选举。完成了１４０个乡镇社区文化法制站的建设，广泛开展了内容丰富的志愿者活动、送温暖活动和“市民论坛”的教育活动。丰富多彩的文明创建活动，弘扬了道德风尚，提高了市民素质，为我市全面建设小康社会提供了强大的精神动力。

　　（六）人民生活水平逐步提高，社会保障体系不断完善。

　 　城市居民和农村居民人均可支配收入分别达９９００元和３６３６元，分别增长９.８％和７.４％。税费改革减轻农民负担１.３亿元。年末城乡居民储蓄余额为７０４亿元。住房、汽车、旅游、信息服务等新兴消费逐步升温。城市和农村居民人均住房面积分别达１１.８平方米和４６.２平方米。新型农民合作医疗制度试点工作取得初步成效。在提高城乡生活水平的同时，注重解决社会弱势群体的实际困难，农村特困户救助和扶贫对象达３６.９万人。全年转移农村劳动力７２万人，劳务收入达４２.３亿元。启动了农村五保老人集中供养试点，新建了一批敬老院。建立了城市特困群体的救助制度。认真落实扩大就业的优惠政策，新增就业岗位８.１万个。下岗职工基本生活保障向失业保险并轨工作基本完成。社会保障覆盖面不断扩大，离退休职工养老金足额发放，参加基本养老保险、医疗保险、失业保险人数分别达５５.８万人、６２万人、４９.４万人。建立和完善低保动态管理制度，年末低保人数达１１.４万人。棚户区居民的住房条件得到改善。

　　 各位代表，２００３年是我市改革开放取得新的进展、经济综合实力和发展后劲显著增强的一年；是城乡面貌发生可喜变化、人民生活水平明显提高的一年。发展令人鼓舞，成就催人奋进。这是市委正确领导的结果，是市人大、市政协有力监督、支持的结果，是全市人民共同努力、付出心血和汗水，广大干部尽心尽责、任劳任怨地工作，各个方面给予热情帮助和大力支持的结果。在此，我代表市人民政府向全市人民、驻长解放军、武警官兵和政法干警，向各民主党派、工商联、无党派人士、各人民团体，向所有关心和支持长沙发展的海内外朋友表示衷心的感谢，并致以崇高的敬意！

　 　回顾过去的一年，我们清醒地看到，在发展过程中还存在不少问题和困难。主要是国民经济整体素质还不高，尤其是工业化发展水平和规模效益亟待提高；农村基础仍然薄弱，农民增收难度大；就业、社会保障和扶贫解困的任务艰巨；公共卫生、生态保护、环境治理、社区建设等问题都需要高度重视；维护稳定和安全生产的压力很大；行政工作效率尚需进一步提高。所有这些，在今后的工作中必须采取有力措施，认真加以解决。

　　二、２００４年政府工作的总体目标和主要任务

　 　２００４年是我市加快发展速度，提高发展水平，增强发展后劲，“大干新三年，再创新辉煌”的起始之年。必须审时度势，把握全局，只争朝夕，乘势而上。

　　 今年政府工作的总体要求是：深入贯彻落实党的十六大和十六届三中全会精神，紧紧围绕全面建设小康社会的总体目标，坚持以人为本，树立全面、协调、可持续的发展观，深化改革，扩大开放，统筹发展，维护稳定，进一步完善社会主义市场经济体制，加快推进工业化、城市化、农业产业化进程，努力提高人民生活水平，实现国民经济持续快速协调健康发展和社会全面进步。今年全市经济和社会发展的主要预期目标是：国内生产总值增长１２％以上；全社会固定资产投资总额增长１８％以上；社会消费品零售总额增长１３％以上；地方财政收入增长１５％以上；城市居民和农村居民人均可支配收入分别增长９％和７％；城镇登记失业率控制在４％以内；人口自然增长率控制在５‰以内。

　　 按照上述总体要求和工作目标，今年要着力抓好以下八个方面的工作：

　 　（一）突出发展产业集群，全力推进新型工业化进程。要继续实施“兴工强市”战略，把握市情，坚定不移地走工业化带动城市化和农业产业化的发展之路。力争实现工业增加值３２０亿元，增长１５％。一是培植工业核心增长极，实现产业向园区聚集。着力构筑以高新区、经开区两个国家级工业园区为龙头的工业园区产业带，促进企业集群式发展，形成工业经济核心增长极。实现高新技术产业产值４９０亿元，增长２５％以上。二是壮大支柱型产业，凸现标志性工程的带动效应。进一步提升电子信息、装备机械、烟草食品三大支柱产业优势，加快十大标志性工程企业和集团的发展，使其在规模工业总产值中的比重分别达到５０％和５５％。继续推进品牌战略，打造全国知名品牌和驰名商标，把“长沙制造”转化为优质产品的代名词。三是加大产业投入，推进产业结构优化升级。完成技改投入和工业基建９６亿元，增长２０％。着力抓好韩国玻壳、中科软件、湘江涂料、青岛啤酒、湘计算机、中联重科、三湘客车、三一重工、长丰二期等重大技改和基建项目。利用高新技术和先进适用技术改造传统产业，实现高新技术产业和传统产业协调发展。四是做好配套服务，构建工业发展支持体系。认真落实我市加快工业发展的政策，增加各级财政投入，增大产业发展的自主投入。实现产业合作，形成原材料供应、生产加工、市场营销一体化产业格局。继续扶持能够提供就业岗位，有竞争优势的劳动密集型企业。推进投融资市场的多元化，为企业发展提供更大的资金支持。五是加大工业企业的内扩外联力度。通过招商、资产重组、强强联合、收购兼并、股份合作等方式，主动承接沿海地区的产业转移，拓展先进制造业发展空间。在重点发展壮大一批规模较大和实力雄厚的大企业、大集团的同时，加强产业配套。鼓励中小企业利用自身优势与大企业、大集团形成协作配套关系，发展一批专业化程度较高的中小企业，拓展延伸产业链，真正突出和发挥工业对整个经济的带动效应。

　 　（二）着力繁荣农村经济，加快城乡一体化步伐。要始终坚持把农业、农村和农民问题作为全局工作的重中之重，紧紧围绕增加农民收入，加快农业和农村经济发展。完成农业总产值、乡镇企业产值１４３亿元和９５８亿元，分别增长５％和１４％。农村居民人均可支配收入达３８９０元。一是加大农村公共投入，改善农村生产生活条件。坚持统筹城乡发展，加大对农村教育、卫生、交通、电网、水利设施等公共事业的投入。全市改建县乡公路３８０公里，完成水利设施建设投资１０亿元，新建沼气池４万个。继续抓好人畜饮水、农村能源以及退耕还林、退田还湖等项目实施。二是调整优化农业结构，加强农业技术推广工作。突出抓好隆平高新科技园和其他农业科技园建设。在稳定粮食生产能力的前提下，继续调整优化种养业结构，发展高附加值的经济作物、特色养殖业和农产品加工业。着力提升优质茶、优质稻、放心菜、中药材四大优势产业。整合农业资源，发展农村合作经济组织。抓好花卉苗木、茶叶、水产、蔬菜、烤烟、药材、水稻基地建设。壮大农业产业化龙头企业，创造名牌农产品，提高市场占有率，继续办好农博会。加强动物防疫，推行农业标准化生产。加大农产品商标注册和绿色食品、有机食品认证工作的力度，提高农产品的市场竞争力。三是建立城乡劳动者平等就业制度，加快农民向非农产业转移。发展农村劳务经济，提高劳务输出的组织化程度，拓宽农民增收渠道。要着力解决拖欠民工工资等实际问题。加强农村规划和加快小城镇建设。提升乡镇企业发展水平，办好中小企业工业园，完善中小企业贷款担保机制。我们要更加关注农村，关心农民，支持农业，做到上下联动，形成共识，满怀感情，一以贯之，把解决“三农”问题作为全面建设小康社会的重点来抓，全面开创农村工作新局面！

　 　（三）继续推进城市建设，创造良好的人居环境。一是以建设新区为重点，不断优化拓展城市发展空间。按照修编后的城市总体规划，突出河西科教新城和天心生态新城的建设。高标准改扩和建设岳麓大道、远大路、韶山路二期、黄兴北路、人民路延长线等城市道路。全年投资８５亿元，完成重点项目５６个。加快发展浏阳、宁乡两大卫星城市，促进区域内基础设施的共建共享。二是以创造最佳人居环境为目标，促进人与自然和谐发展。重点保护好岳麓山风景名胜区和桔子洲。建设生态、资源节约型城市，切实保护水资源、土地、森林资源，启动引水及水质环境工程、长沙电厂、抽水蓄能电站和城市生态圈建设，增强可持续发展能力。积极开展创建国家园林城市活动，在全市广泛开展植树造林，新增绿化面积１０５公顷，其中城区新增公共绿地１００万平方米。推动城市综合整治向纵深发展，改造１００条背街小巷。推进社区物业管理，创新“门前三包”综合整治模式。三是以先进文化为指导，推动历史文化名城建设。

　　在城市建设和棚户改造中更加注重历史街区和历史文物的保护和维护工作。重点保护和建设好开福寺宗教文化风貌区和太平街、潮宗街、清水塘等历史文化街区。四是以一山一洲两岸为核心，重点建设湘江滨水区，保护和适度开发浏阳河滨水区。启动和加快桔子洲、滨江风光带、滨江标志性建筑和三叉矶大桥的建设，续建湘江路南北延长线。用三年的时间建设好市博物馆、图书馆和滨江音乐厅。强化规划的龙头作用，搞好城市设计，精心组织建设。集中群众智慧，全力打造充分聚淀和彰显大自然的神韵、山水的灵秀、厚重的历史文化底蕴与现代文化精华，饱含长沙人民的理想追求、极富个性和魅力、独具山水洲城特色的城市景观，形象而丰富地展示长沙的天然之美、人文之美。

　　 （四）全面深化改革，增强经济和社会发展活力。一是深化国有企业改革。年内要基本完成国有企业改制改造，完善法人治理结构。着力推进企业重组、兼并、破产，大力发展混合所有制经济，使股份制成为公有制的主要实现形式。形成一批主业突出，核心竞争力强的企业或企业集团。继续加大对非公有制经济的扶持力度，多种形式支持中小企业健康发展，形成各类企业优势互补，专业分工明确，社会协作紧密的企业发展新格局。二是推进国有资产管理体制改革。建立政府公共管理职能与国有资产出资人职能分开的制度。全市国有资产要分类统计，摸清家底，建立制度，实施有效管理。严格审核国有改制企业的资产处置，加强对国有产权转让的监督，完善产权交易机制，确保产权、股权公开交易、有偿转让，国有资产保值增值。加快建立归属清晰，权责明确，保护严格，流转顺畅的现代产权制度。三是推进公用事业改革。引入市场竞争机制，打破行业垄断，引导非公有制经济参与公用事业的改制、经营，形成多元投资主体共同运营的竞争格局。四是深化投融资体制改革。要进一步确立企业的投资主体地位，政府主要通过规划、政策扶持、产业指导和信息发布引导社会投资方向。推行以公司为主体的城市投资建设管理模式。国有投资公司要实行股份制改造，壮大投资实力。完善政府债务管理办法，壮大偿债基金规模。经营性城市资产要实行完全的市场运作，准经营性资产要剥离管理职能迅速走向市场，非经营性资产要引入竞争机制，提高使用效率。五是深化农村体制改革。全面落实农村税费改革政策，取消农业特产税，农业税税率降低一个百分点。财政对种粮农民实行直接补贴。要把减轻农民负担与乡镇机构、农村教育体制配套改革结合起来；完善县乡财政体制，减少财政供养人员，抓好乡镇消赤减债工作；精简村组干部，优化教师队伍。完善土地流转制度。逐步发展新型农民合作医疗。搞好农村金融体制改革，加大信贷支农力度。抓好农村全面小康示范村建设。六是加快培育以资本、土地、技术、劳动力市场为重点的要素市场改革。加快培育拟上市公司，做好上市公司的增发、配股等再融资工作。严格界定经营性和公益性建设用地，使土地得到最有效的利用。加大知识产权保护和转化力度。加快人才、技术市场建设，打破一切限制生产要素合理流动的体制性障碍。要努力营造鼓励人才干事业，支持人才干成事业，帮助人才干好事业的社会环境，让一切劳动、知识、技术、管理和资本的活力竞相迸发，让一切创造财富的源泉充分涌流！七是推进财政体制改革。坚持依法治税理财，着力加强财源建设，严格税收征管，优化支出结构，初步搭建公共财政框架。加大对公共服务和社会保障的投入，新增的公共事业支出应主要向农村倾斜，逐渐增加对困难地区的财政转移支付。落实出口退税改革政策，继续抓好各项财政管理制度的落实。实行部门和项目预算制，将专项资金细化到项目。投入到经营性项目的财政资金要实行有偿使用。要进行财政支出绩效考核，提高财政资金的使用效益。八是深化行政体制改革。减少审批事项，凡是通过市场机制能够解决的问题，都由市场机制去解决；通过市场机制难以解决的，要通过规范、公正的中介机构来解决；确需保留行政审批的，也要简化程序，缩短审批时间，提高服务效率。完善统计改革，提高宏观经济管理水平。

　　 （五）发展壮大服务业，全面扩大对外开放。一是优化商贸流通布局，注重发展新型业态。社会消费品零售额要达到５１０亿元。鼓励市内同一业态或经营内容相近的连锁企业，整合资源，实现低成本扩张。建设多层次物流配送体系，高起点规划建设好金霞物流园。支持有条件的企业发展电子商务。二是壮大服务支柱产业，着力发展会展经济和文化产业。争创首批中国最佳旅游城市。构筑旅游产业链，完成旅游总收入１４０亿元，增长１９％。加快发展会展业，形成长沙会展品牌。要发挥我市文化、体育设施较为完善的优势，在文化产业发展上取得新的突破。稳步发展房地产业，激活房地产二级市场。大力抓好现代信息、中介服务、社区服务等新型服务业。三是大力发展外向型经济，全面提高对外开放水平。办好“国际友城经贸合作洽谈会”和“长沙（香港）招商暨经贸洽谈会”。直接利用外资６亿美元，引进市外境内资金２８０亿元。提高项目招商、网上招商、园区招商、代理招商的水平，建立以实际到位资金为主的目标考核体系。努力提高出口商品质量、档次和附加值，全面提高出口商品的竞争力。充分发挥外事侨务的桥梁作用，加强国际文化、经济和民间的合作与交流，扩大长沙知名度。

　 （六）坚持以人为本，促进社会事业全面发展。一是优先发展科技教育事业。充分发挥各类科技园区、大学科技园的科技孵化、成果转化作用，构建区域科技创新体系。抓好教育体制、管理模式、教学方式的改革，提高素质教育水平，完善义务教育体系。加大教育投入，优化教育资源配置，推进各类教育均衡发展。发展民办教育，加强继续教育、社区教育和职业教育。二是加快文化卫生体育等事业发展。发展壮大长沙晚报报业集团、长沙广电集团。抓好新闻出版、艺术院团、电影放映的体制改革。承办好中国首届电影音乐颁奖会。加强公共卫生设施建设，建立健全疾病预防控制体系和医疗救治体系。稳定低生育水平，提高人口素质。完善卫生执法监督体系，加强环境卫生监测。广泛开展群众体育和健身活动，提高竞技体育水平。做好奥运会亚洲区中韩足球预选赛的各项筹备工作。三是要做好就业和再就业工作，完善社会保障体系。就业是民生之本，也是安国之策。各级政府要切实履行职责，时刻把抓好就业这件大事放在心上，落实在工作中。要大力发展劳动密集型企业、非公有制企业，千方百计扩大就业，全市要新增就业岗位８万个。完善就业再就业培训和服务体系，加大再就业援助力度，提高劳动者就业技能，做好劳务输出的组织、服务工作。保障离退休人员养老金、下岗失业人员救济金、城市居民最低生活保障金的及时足额发放。完善多层次保障体系，加大对贫困地区和困难群体的扶持力度。要始终把人民群众的安危冷暖当作第一信号，始终把实现、维护、发展人民群众的利益当作第一选择，始终把为人民群众解决实际困难和问题作为对我们工作的最好慰勉！

　　 （七）优化发展环境，加强民主法制建设。一是完善民主制度，加强法制建设。各级政府要自觉接受人大及其常委会的监督，执行人大决议，定期报告工作。支持人民政协履行政治协商、民主监督和参政议政的职能。加强与各民主党派、工商联和无党派人士的联系，充分发挥工会、共青团、妇联等人民团体的桥梁和纽带作用。加大“四五”普法力度，不断提高干部群众和青少年的法律意识。加强基层民主法制建设，保证人民群众依法直接行使民主权利，完善村民自治、居民自治。二是加大文明创建力度，营造积极向上的社会氛围。加强社会公德、职业道德和家庭美德教育。广泛开展市民论坛、社区教育等各种群众性精神文明创建活动，力争再获“全国社区建设示范市”的荣誉称号。建立信用监督和失信惩戒制度，倾力打造信用长沙，实行社会联动措施，使失信者寸步难行，进退维艰；使诚信者正气弘扬，蔚然成风。加强国防教育和“双拥”工作，提高创建“双拥模范城”的工作水平。三是从严规范经济秩序，着力维护社会稳定。完善市场监管体系，严厉打击制假售假、商业欺诈等违法行为，坚决惩治医药购销中的不正之风，查处乱收费行为。对损害发展环境的人和事，既要追究当事人的责任，又要追究部门领导的责任，绝不姑息迁就，绝不放任不管，绝不搞下不为例。加强思想疏导工作，正确处理好人民内部矛盾。强化社会治安综合治理，落实好维护稳定的责任制。深入开展“严打”整治斗争。重视民族宗教事务，加强信访、仲裁、复议和人民调解工作。及时妥善处理拆迁安置、企业改制、劳资等方面的矛盾纠纷。狠抓安全生产，杜绝特大安全事故发生，确保人民群众生命财产的安全和社会稳定。

　 　（八）加强学习，转变职能，建设务实清廉可问责的服务型政府。一是要创新政府运行机制和管理方式。将政府职能切实转到经济调节、市场监督、社会管理和公共服务上来。坚决把属于市场的事交给市场，把属于社会的事交给中介组织，真正把政府经济管理职能转到主要为市场主体服务和改善环境上来。坚决做到政企、政资、政事分开，把更多的精力用于社会管理和公共服务。重点抓好就业和再就业，城乡居民收入增加，农民减负，县乡公路建设，生态环境保护，公共卫生建设，城乡扶贫解困和困难家庭子女入学，保护民工合法权益和建设农民工廉租房等８件顺民意得人心的实事。二是要坚持依法行政和推行政务公开。要认真贯彻实施《行政许可法》，积极探索与市场经济体制相适应的行政执法方式和手段，要公正执法，严格实行执法责任制和执法过错追究制。要在更大范围推行政务公开，把政府行使事权、人权、财权的行为置于广大人民群众的监督之下，让人民群众拥有更大、更广泛的知情权和监督权。各级政府要率先遵守信用规则，做诚信的模范和带头者。三是要勤政廉政，从严治政。“政之所兴，在顺民心；政之所废，在逆民心”。每一个政府工作人员，都要坚持学习、升华境界、善于思考，勤于政事、廉于政务、甘于清贫、乐于奉献，真正做到权为民所用、情为民所系、利为民所谋。常察民之疾苦、常思民之所忧、常行富民之举、常责负民之过。任何时候都不弄虚作假，欺上瞒下，不提不切实际的口号，不搞劳民伤财的形象工程，不干违民意损民力伤民心的事。坚持制度管人，从严惩治腐败，反对贪图享受，制止挥霍浪费，杜绝形式主义，提倡勤俭节约，忠实履行职责，层层落实问责。真正以不骄不躁、艰苦奋斗、务实求真的工作作风，实实在在的工作业绩，一心一意为人民服务的良好形象，赢得广大人民群众的拥护、支持和爱戴！

　　 各位代表，新的一年是胜利完成“十五”计划，奠定前进的坚实基础，加快发展的关键之年。让我们坚持以“三个代表”重要思想为指导，全面贯彻落实党的十六大精神，在市委的领导下，万众一心，奋发图强，顽强拼搏，勇攀高峰，满怀豪情地在全面建设小康社会的征途上，去创造新的业绩，实现新的跨越！
